# An Instance of Hallucination in a Fowl

**Published:** 1880-04

**Authors:** E. C. Seguin


### II.—AN INSTANCE OF HALLUCINATION IN A FOWL.

By E. C. SEGUIN, M. D.

In the autumn of 1868, while serving as Post Surgeon at Fort Craig, in the territory of New Mexico, I was a witness to the following remarkable example of hallucination and delusion in an animal: The animal was a young chicken, one perhaps three or four months old. A hen had been killed on the morning of the same day, and her head, with a short piece of bloody neck, lay in the court behind our mess quarters. I saw the chicken run up to this lifeless hen, seize its comb in his beak and make unmistakable attempts to "mount." He made frantic efforts to copulate with the lingering body of the hen, which in his fury he was shaking wildly about, over quite a part of the yard. At the same time appropriate movements of his hinder parts were made, just as if he were having connection with an actual hen. The struggle continued for many minutes, apparently much to the fatigue of the young cock.

I then drew two conclusions from the event, which I still think correct, though I am glad to submit them to those who know more than I do of animal psychology.

1. There was hallucination and delusion. The chicken fancied that he saw and felt a real objective hen, and he accepted the illusion as real; there was no correction by judgment.

2. The chicken was under the influence of strong sexual excitement; a state, perhaps, corresponding to what is called satyriasis in man.

